# Metformin promotes innate immunity through a conserved PMK-1/p38 MAPK pathway

**DOI:** 10.1080/21505594.2019.1706305

**Published:** 2019-12-28

**Authors:** Yi Xiao, Fang Liu, Sanhua Li, Nian Jiang, Changyan Yu, Xinting Zhu, Ying Qin, Jing Hui, Lingjie Meng, Changwei Song, Xiao-Fei Li, Yun Liu

**Affiliations:** a Guizhou Provincial College-based Key Lab for Tumor Prevention and Treatment with Distinctive Medicines, Zunyi Medical University, Zunyi, Guizhou, China; b Research Center for Medicine & Biology, Zunyi Medical University, Zunyi, Guizhou, China; c College of Basic Medicine, Zunyi Medical University, Zunyi, Guizhou, China

**Keywords:** Metformin, innate immunity, p38 MAPK pathway, *C. elegans*

## Abstract

Metformin, as the first-line oral drug for type 2 diabetes, has proven benefits against aging, cancer and cardiovascular diseases. But the influence of metformin to the immune response and its molecular mechanisms remain obscure. Metformin increases resistance to not only the Gram-negative pathogens *Pseudomonas aeruginosa* and *Salmonella enterica* but also the Gram-positive pathogens *Enterococcus faecalis* and *Staphylococcus aureus*. Meanwhile, metformin protects the animals from the infection by enhancing the tolerance to the pathogen infection rather than by reducing the bacterial burden. Through the screening of classical immune pathways in *C. elegans*, we find metformin enhances innate immunity through p38 MAPK pathway. Furthermore, activated p38/PMK-1 by metformin acts on the intestine for innate immune response. In addition, metformin-treated mice have increased resistance to *P. aeruginosa* PA14 infection and significantly increased the levels of active PMK-1. Therefore, promoted p38/PMK-1-mediated innate immunity by metformin is conserved from worms to mammals. Our work provides a conserved mechanism by which metformin enhances immune response and boosts its therapeutic application in the treatment of pathogen infection.

## Introduction

With the development of antibiotic-resistant bacteria pathogens, identification of a chemical or pharmacological drug that could influence human immune response and decrease the risks of pathogen infection has become a key goal of innate immunity research. Metformin, an antiglycemic biguanide drug and the first-line drug for the treatment of type 2 diabetes, has been proven to extend lifespan in *Caenorhabditis elegans* [1–5] and mice [6,7]. Furthermore, metformin has been reported to the benefit of the cardiovascular diseases in patients [8]. Metformin also has been used in the treatment of metabolic syndrome and cancer [9,10].

Recent studies have shown that metformin has antimicrobial properties for the treatment of pathogen infection, including *Trichinella spiralis* [11], *Staphylococcus aureus* [12], *Pseudomonasaeruginosa* [13,14], *hepatitis B virus* [15], *hepatitis C virus* [16,17], and human immunodeficiency virus [18]. For example, Calu-3 airway epithelial cocultured cells with metformin treatment inhibited the basolateral glucose-induced apical *P. aeruginosa* growth. Treatment with metformin increased the expression of claudin-1 protein and occludin, which were reduced by *P. aeruginosa* infection [13]. In addition, hyperglycemic mice with metformin treatment led to a significant reduction of airway *P. aeruginosa* bacterial load and a decrease in airway glucose concentrations [14]. Although the mechanism of underlying metformin’s metabolic effects not fully understood, it had been widely attributed to AMPK activation [13,19–21]. Furthermore, metformin inhibited the mitochondrial complex I activity against drug-resistant strains of tuberculosis [22–24]. In addition, metformin decreased the mitochondrial H_2_O_2_ emission in the skeletal muscle of obese rats [25]. Overall, despite the beneficial functions for metformin in multiple cellular processes, its contribution to innate immunity in animals is unknown.

The innate immune system represents the first line of defense against invading pathogens and it is evolutionarily conserved from worms to mammals [26]. During infection, the innate immune system is activated, resulting in antimicrobial response to invading pathogens [26–30]. *Caenorhabditis elegans* has been developed as a valuable genetic model for research on the animal immune response. Through using this tractable model, researchers uncover several signaling pathways that have important roles in controlling the innate immunity, such as the PMK-1/p38 MAPK pathway [31,32], the DAF-2/DAF-16 pathway [33], the MPK-1/ERK MAPK pathway [34], the protein kinase D DKF-2 [35], the G protein-coupled receptor FSHR-1 36, and the G protein GqαEGL-30 [37].

In this study, we investigated the ability of metformin to modulate *C. elegans* host defense. Through the screening of classical immune pathways in *C. elegans*, we found that metformin protects the host against pathogens through governing p38/PMK-1 activation. In addition, p38/PMK-1 functioned in the intestine for innate immunity activation by metformin. This ancient metformin response pathway was conserved from worms to mammals. These findings revealed that the metformin-promoted innate immunity would significantly boost its application to improve the patients of pathogen infection.

## Results

### Metformin enhances pathogen resistance

**Figure 1.**
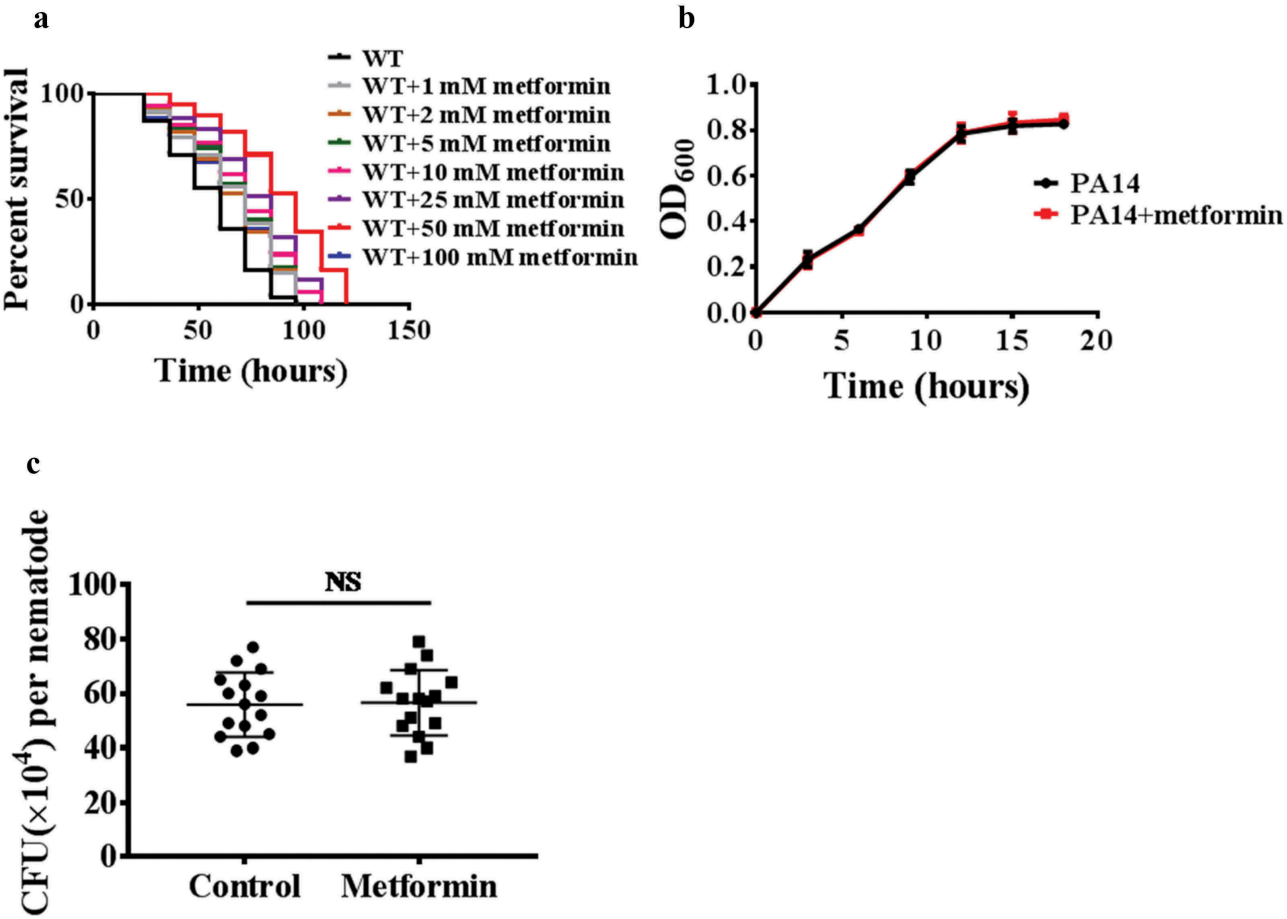
Metformin enhances pathogen resistance. (a) Metformin promotes innate immune response to *P. aeruginosa* PA14 compared to WT in a dose-dependent manner (*P< 0.05, log-rank test). (b) metformin (50 mM) did not delay the proliferation of *P. aeruginosa* PA14. (c) Metformin (50 mM) did not affect the colony-forming units (CFUs) of bacteria in WT worms after *P. aeruginosa* PA14 infection. These results are mean ± SD of three independent experiments, each involving 15 parallel groups. NS, no significance.

To determine whether metformin promotes the innate immunity, worms were exposed to the human opportunistic pathogen *Pseudomonas aeruginosa*(PA14), and we found that wild-type animals treated with metformin (1 mM, 2 mM, 5 mM, 10 mM, 25 mM, 50 mM, and 100 mM) exhibited increased resistance to *P. aeruginosa* in dose-dependent manner (Figure 1(a); Table S2). Like lifespan [2], metformin shows a saturating effect on pathogen resistance, maximal at 50 mM drug, and declining at 100 mM drug (Figure 1(a); Table S2). After metformin treatment, worms exposed to *Staphylococcus aureus, Enterococcus faecalis*, or *Salmonella enterica* have a higher survival rate (Figure S1A, 1B, and 1C; Table S3). These results suggest that metformin enhances the innate immunity in *C. elegans*. Previous study has shown that metformin inhibits *E. coli* proliferation through a dose-dependent manner [2]. To test whether metformin promotes host immune response via inhibiting the growth of pathogenic bacteria, we used the bacterial growth assay and demonstrated that metformin did not inhibit the proliferation of *P. aeruginosa* PA14 (Figure 1(b)), *S. aureus, E. faecalis, S. enterica* (Figure S2A, 2B, and 2C). Taken together, these results indicate that metformin action on immune response is not caused by inhibition of bacterial growth. *GacA* is a virulence-related gene in pathogen *Pseudomonas aeruginosa* [38]. Worms were exposed to the human opportunistic pathogen *Pseudomonas aeruginosa*(PA14*GacA* mutant), and we found that wild-type animals treated with metformin (50 mM) exhibited increased resistance to *P. aeruginosa* (PA14*GacA* mutant) (Figure S3; Table S3). The clearance of the bacterial load is part of host defense against pathogen infection [39,40]. Thus, we then examined whether metformin influenced the bacterial accumulation. Metformin did not affect the colony-forming units (CFUs) of bacteria in WT worms after *P. aeruginosa* PA14 infection (Figure 1(c)). Overall, these results suggest that metformin enhances host tolerance to pathogen infection rather than reduces the bacterial burden.

### Metformin promotes innate immunity through the p38 MAPK pathway

**Figure 2.**
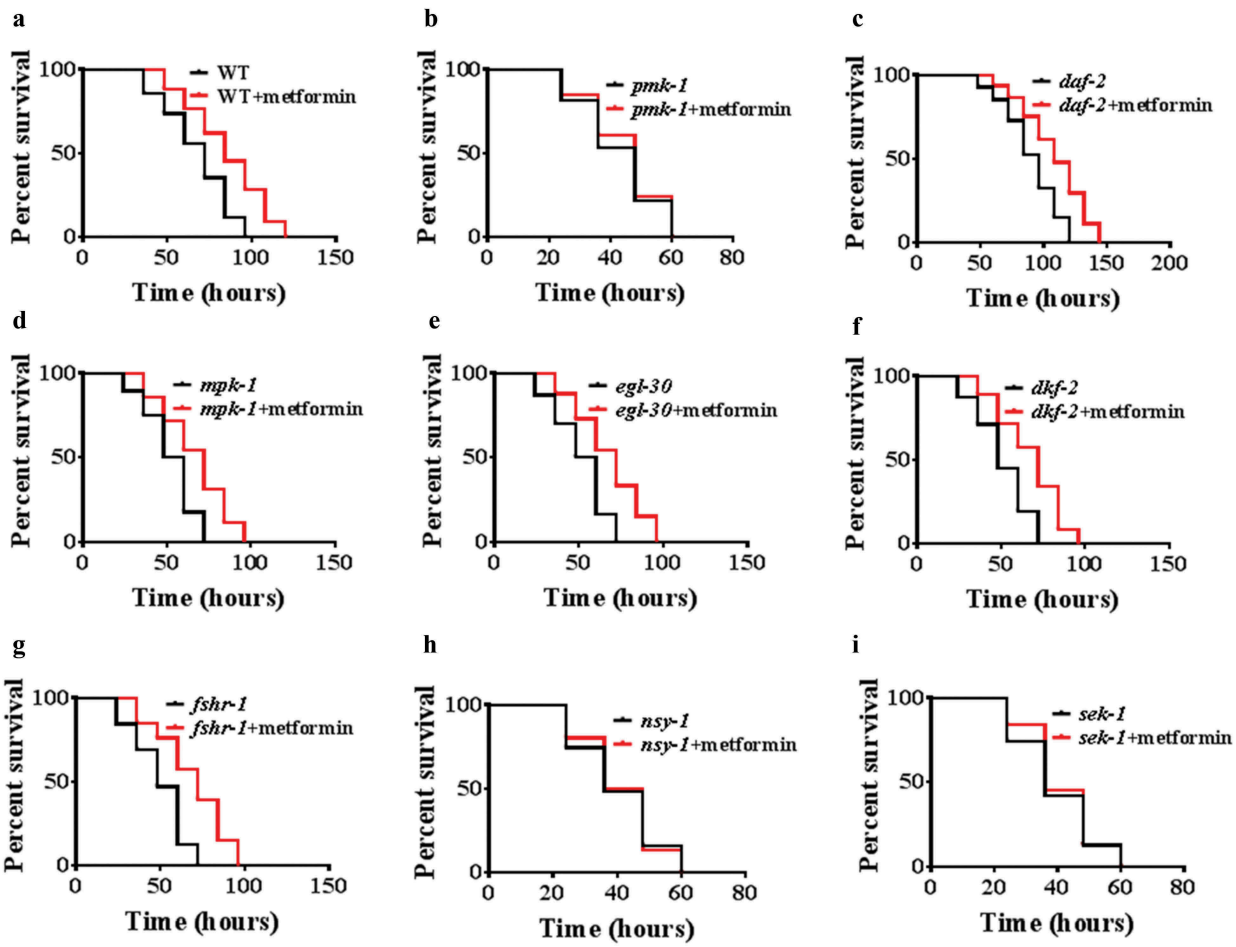
Metformin promotes innate immunity through the p38 MAPK pathway. (a–i) PMK-1/p38 MAPK is involved in metformin-mediated innate immunity. Metformin (50 mM) enhanced resistance to *P. aeruginosa* PA14 in WT (N2) (a), *daf-2(e1370)* (c), *mpk-1(n2521)* (d), *egl-30(n686)* (e), *dkf-2(ok1704)*(f), *fshr-1(ok778)*mutants (g), but not in *pmk-1(km25)* mutants (b). *P< 0.05 versus worms + metformin (log-rank test). (h and i) Mutations in the components of the p38 MAPK pathway suppressed metformin-mediated resistance of worms to PA14. (h) *nsy-1(ag3)*; (i) *sek-1(ag1)*.

To investigate the molecular mechanisms by which metformin confers protection against pathogens, we screened several signaling pathways, which involved in innate immunity in *C. elegans*, such as p38 MAPK/PMK-1 [31], DAF-2/DAF-16 [33], ERK MAPK/MPK-1 [34], EGL-30 [37], protein kinase D/DKF-2 [35], and FSHR-1 [36]. We found that metformin failed to enhance resistance to *P. aeruginosa* PA14 infection in *pmk-1(km25)* mutants, compared with WT worms (Figure 2(a, b); Table S2). However, metformin promoted the survival rates of *daf-2(e1370), mpk-1(n2521), egl-30(n686), dkf-2(ok1704)*, and *fshr-1(ok778)* mutants after *P. aeruginosa* PA14 infection (Figure 2c-g; Table S2). Furthermore, we then tested the core components of the p38 MAPK signaling, the MAPK kinase NSY-1 and the MAPK kinase SEK-1. We found that metformin could not confer resistance to PA14 infection in *nsy-1(ag3)* and *sek-1(ag1)*mutants (Figure 2(h, i); Table S2). VHP-1 MAPK phosphatase (MKP), a homolog of mammalian MKP7, also regulates pathogen resistance through the modulation of PMK-1 activity [41]. *Vhp-1*RNAi was non-additively beneficial with metformin (Figure S4A; Table S3). Furthermore, knockdown of *vhp-1*did not further increase the level of *K08D8.5p::GFP*(p38 target gene) under metformin treatment (Figure S4B). Taken together, these results suggest that metformin acts on the VHP-1/p38 MAPK pathway to enhance innate immunity in *C. elegans*.

### Metformin activates p38 MAPK signaling in *C. elegans*

**Figure 3.**
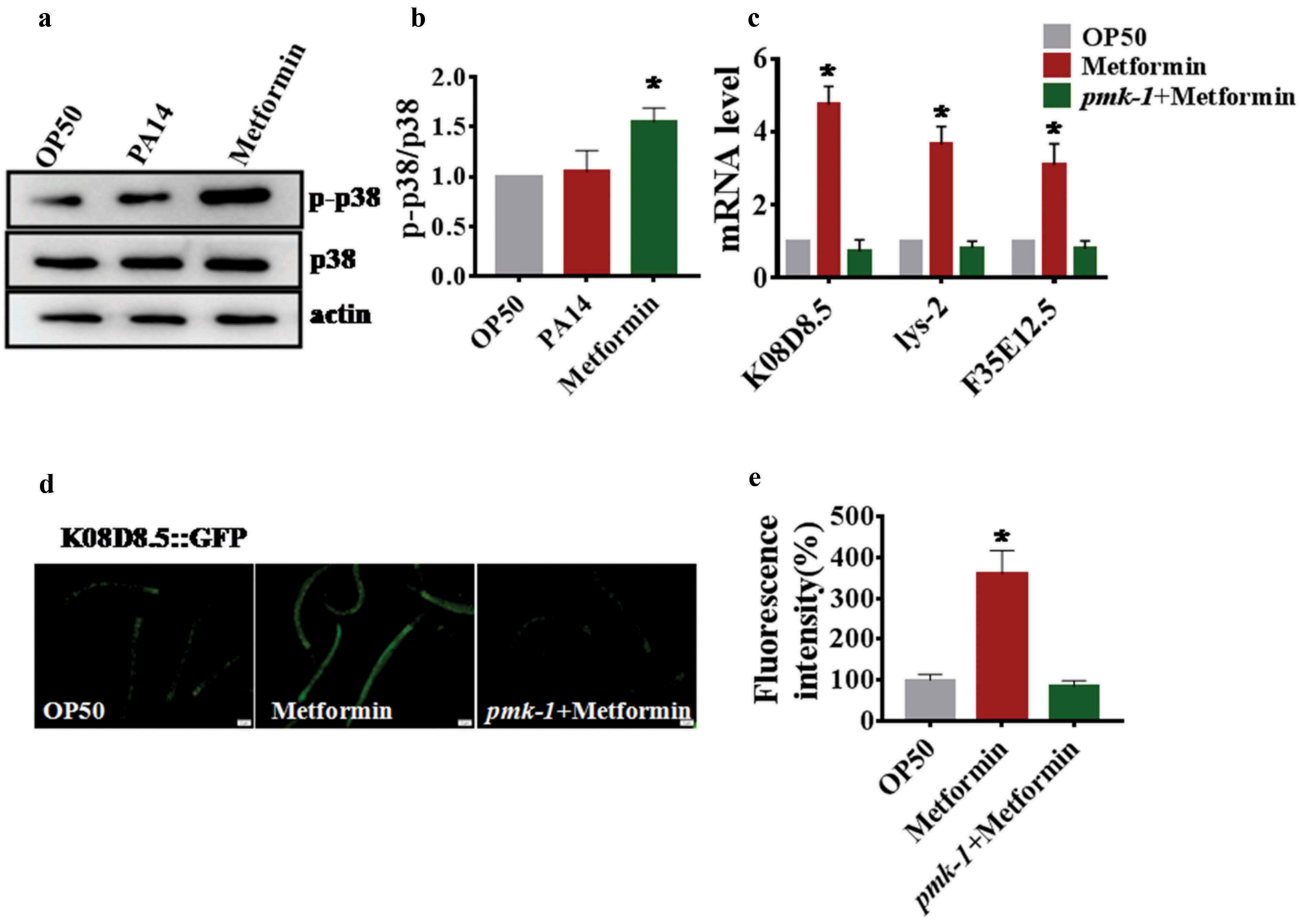
Metformin activates p38 MAPK signaling in *C. elegan*s. (a) The phosphorylation of p38 MAPK was elevated in WT worms (N2) exposed to metformin (50 mM). (b) The right panel shows quantification of phosphorylated p38 MAPK levels. These results are mean ± SD of three independent experiments performed in triplicate. *P< 0.05 vs *E. coli*OP50 (one-way ANOVA followed by a Student-Newman-Keuls test). (c) The mRNA levels of three p38 MAPK targets *K08D8.5, lys-2,* and *F35E12.5* in worms exposed to metformin (50 mM). These results are mean ± SD of three independent experiments performed in triplicate. *P< 0.05 versus OP50 (one-way ANOVA followed by a Student-Newman-Keuls test). (d) Expression of *K08D8.5p::GFP* was up-regulated in WT worms, but not in worms subjected to *pmk-1*(km25) mutants, exposed to metformin (50 mM). (e) The right panel shows quantification of fluorescence intensity. These results are mean ± SD of three independent experiments performed in triplicate. *P< 0.05 versus OP50 (one-way ANOVA followed by a Student-Newman-Keuls test).

To determine whether metformin activates p38 MAPK signaling or not, we tested the level of phosphorylation PMK-1, which is a signature of its activation [35,39]. We found that *P. aeruginosa* PA14 infection did not heighten the phosphorylation levels of p38 MAPK in *C.elegans* (Figure 3(a,b)), which is consistent with a recent study [42]. In contrast, metformin significantly increased the protein levels of active PMK-1 in *C.elegans*(Figure 3(a,b)). Next, we tested the expression of PMK-1-targeted genes, *K08D8.5, lys-2*, and *F35E12.5* [43]. Quantitative real-time PCR analysis demonstrated that PMK-1-dependent genes were up-regulated in metformin-treated animals compared with OP50 (Figure 3(c)). However, metformin failed to enhance their expression in the *pmk-1(km25)*worms (Figure 3(c)). Furthermore, we detected the expression of *K08D8.5* through using the transgenic worms expressing *K08D8.5p::GFP*. We observed higher levels of GFP in metformin-treated animals, but not in *pmk-1(km25)* worms (Figure 3(d,e)). In addition, to find out whether metformin activated the PMK-1 through a canonical metformin target *aak-2, skn-1*, mitochondrial complex I(*gas-1*), mTOR(*let-363*). Using the *K08D8.5::GFP* reporter, we found that knockdown of *aak-2, skn-1, gas-1, let-363* did not influence the level of *K08D8.5::GFP* in metformin-treated animals (Figure S4B and 4C) indicating that the activation of PMK-1 by metformin did not depend on canonical targets. In conclusion, these findings indicate that metformin activates the p38 MAPK pathway in *C. elegans*.

### Intestinal PMK-1 enhances resistance to pathogen infection after metformin treatment

**Figure 4.**
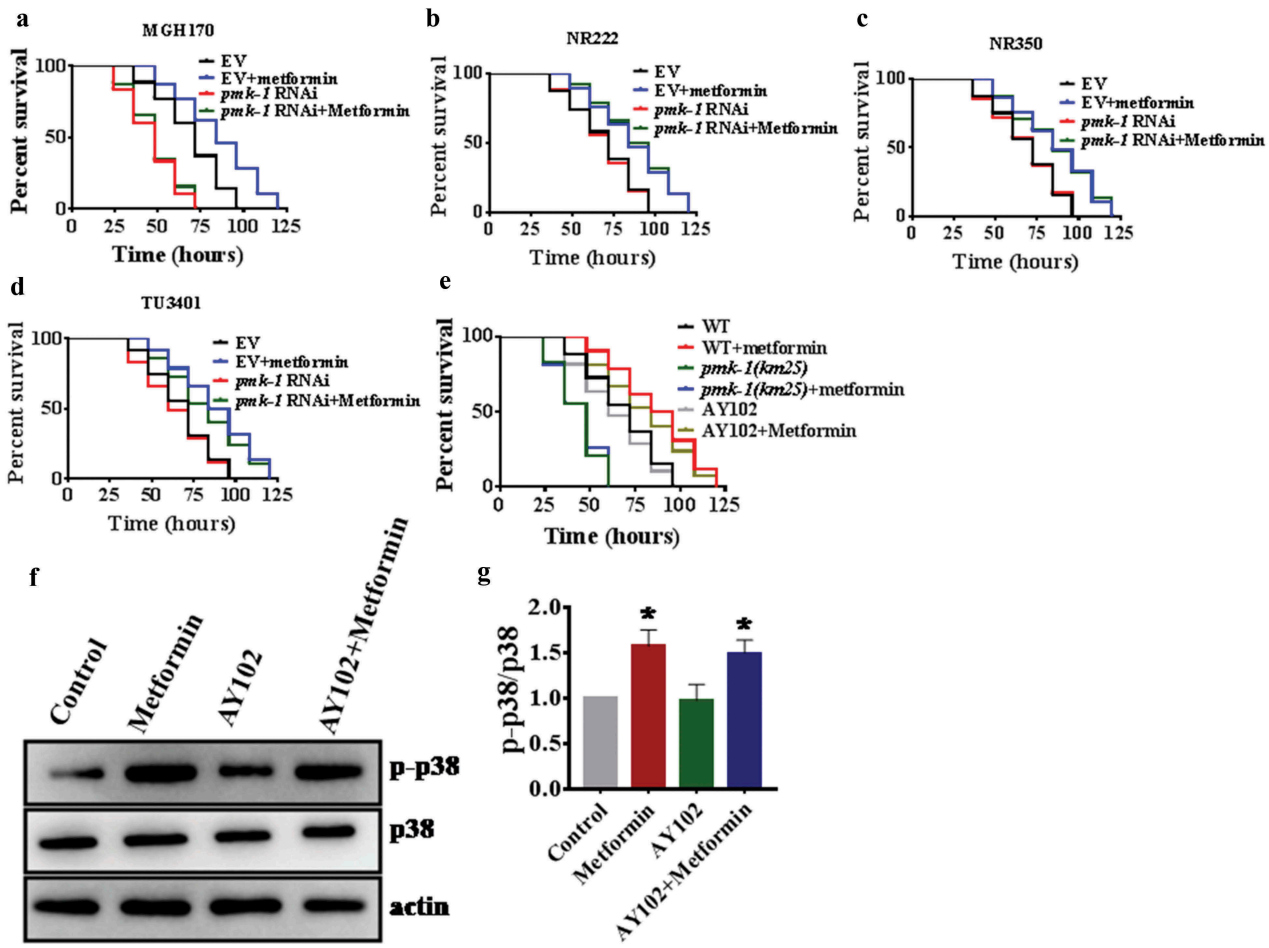
Intestinal PMK-1 enhances resistance to pathogen infection after metformin treatment. (a) metformin (50 mM) did not increase resistance to *P. aeruginosa* PA14 infection in the intestine knockdown of *pmk-1*worms. However, RNAi of *pmk-1* in hypodermis (b), muscle (c), and neuron (d), respectively, after metformin treatment did not prevent metformin action on promoting *pseudomonas* resistance. (e) Expression of *pmk-1* under the intestinal-specific *vha-6* promoter (AY102) restored resistance against *P. aeruginosa* PA14 infection in *pmk-1(km25)* mutants after treatment with metformin (50 mM) .*P< 0.05 versus worms + metformin (log-rank test). (f) Pre-treatment of AY102 worms with metformin (50 mM) restored the level of active PMK-1. (g) The right panel shows quantification of phosphorylated p38 MAPK levels. These results are mean ± SD of three independent experiments performed in triplicate. *P< 0.05 versus OP50 (one-way ANOVA followed by a Student-Newman-Keuls test).

To determine tissue-specific actions of PMK-1 in response to *P. aeruginosa* infection after metformin treatment, we performed tissue of knockdown experiments using MGH170 strain in the intestine [44], NR222 strain in hypodermis [45], NR350 strain in muscle [45], and TU3401 strain in neuron [46]. We found that knockdown of *pmk-1* in the intestine of MGH170 animals completely abolished the protection conferred by metformin (Figure 4(a); Table S2). However, after metformin treatment, other knockdowns did not prevent metformin action on promoting *pseudomonas* resistance (Figure 4(b-d); Table S2). These results demonstrate that metformin- induced immune response is necessary to the intestinal activity of PMK-1. To further determine whether metformin depends on the intestinal PMK-1 to regulate immunity, we utilized the AY102 transgenic strain, which carried *pmk-1* under the control of the intestinal *vha-6* in *pmk-1(km25)* worms. We found that expression of *pmk-1* in the intestine after metformin treatment fully rescued the survival rate of *pmk-1(km25)* animals exposed to *P. aeruginosa* PA14 (Figure 4(e); Table S2). Furthermore, pre-treatment of AY102 worms with metformin restored the level of active PMK-1 (Figure 4(f, g)). Taken together, these results suggest that metformin triggered immunity requires the intestinal activity of PMK-1.

### Metformin protected mice against *P. aeruginosa* infection

**Figure 5.**
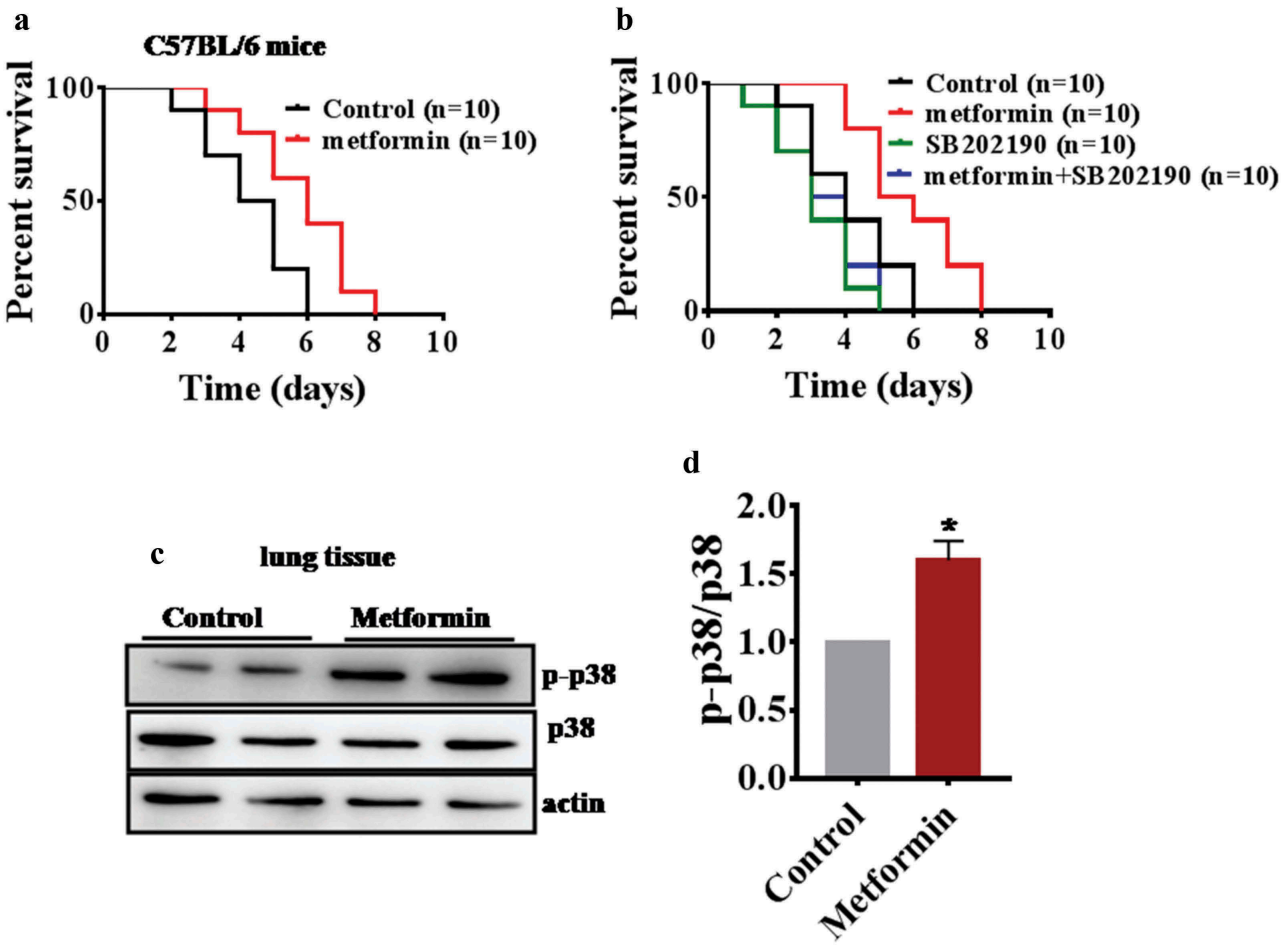
Metformin protected mice against *P. aeruginosa* infection and increased p-p38 in the lung. (a) Metformin (200 mg/kg body weight) treated mice increases the resistance to *P. aeruginosa* PA14 infection compared with control mice. *P< 0.05 (log-rank test). (b) The p38 inhibitor SB202190 increased the susceptibility to *P. aeruginosa* PA14 infection compared with control mice *P< 0.05 (log-rank test) and suppressed the enhanced resistance to *P. aeruginosa* PA14 upon metformin (200 mg/kg body weight) treatment *P =*0.0512 (log-rank test). (c) Metformin (200 mg/kg body weight) significantly increased the levels of active PMK-1 in the lung. (d) The right panel shows quantification of phosphorylated p38 MAPK levels. These results are mean ± SD of three independent experiments performed in triplicate. *P< 0.05 versus control (one-way ANOVA followed by a Student-Newman-Keuls test).

Next, we examined whether metformin enhances the innate immunity in mice. The mice were treated with 200 mg/kg body weight metformin and control mice were subjected to infection (1.0 × 10^6^ CFUs/mouse) with *P. aeruginosa* PA14. The result showed that metformin-treated mice had increased resistance to *P. aeruginosa* PA14 infection compared with control mice (Figure 5(a); Table S4). The p38 MAPK pathway is conserved from worms to mammals. To test whether metformin acts on the p38 MAPK pathway to enhance innate immunity in mice, the treating mice with 200 mg/kg body weight metformin or 20 μg/kg body weight p38 inhibitor SB202190 [47] and control mice were subjected to infection (1.0 × 10^6^ CFUs/mouse) with *P. aeruginosa* PA14. The results showed that SB202190 increased the susceptibility to *P. aeruginosa* PA14 infection compared with control mice (Figure 5(b)). In contrast, SB202190 suppressed the enhancement resistance to *P. aeruginosa* PA14 upon metformin treatment (Figure 5(b); Table S4). Meanwhile, we also found that metformin significantly increased the levels of active PMK-1 in mice (Figure 5(c, d)). Taken together, metformin may also activate immunity in mice through p38 MAPK pathway.

### Discussion

In this study, we identify that preventive application of metformin protects *C. elegans* against Gram-negative pathogens *P. aeruginosa, S. enterica* and the Gram-positive pathogens *E. faecalis, S. aureus*. This effect is associated with a conserved mechanism that regulates the innate immunity in *C. elegans*, including the p38/PMK-1 MAPK pathway. In addition, our results indicate that the enhancement of immune responses by metformin requires intestinal PMK-1 and that it does not protect the worms from the infection by reducing the bacterial burden but rather by making the worms more tolerant to the infection. Furthermore, metformin also activates immunity in mice through p38 MAPK pathway. Taken together, these results uncover an alternative mechanism by which antibiotic action-like metformin may protect the host from bacterial infections.

Previous work has shown that metformin reduced the levels of pro-inflammatory cytokines and improved survival in several endotoxemia models of mice [19]. Furthermore, Metformin was active against *T. spiralis, S. aureus, P. aeruginosa*, and *hepatitis B virus*in vitro [19]. These researches imply that metformin may have a potential role in the therapy for pathogen infection. However, whether the metformin influences the immune response and the underlying molecular mechanisms remain obscure. Here, using model organism *C. elegans*, we investigate the ability of metformin to modulate host defense. Through the screening of classical immune pathways in *C.elegans*, we find that metformin protects the host against pathogens through governing p38/PMK-1 activation. Animal studies also show that metformin protects mice against *P. aeruginosa* infection and it activates the p38/PMK-1. These results presented clearly show that metformin acts as an activator of conserved immune pathways. Intriguingly, metformin may function on AMPK to prevent the *P. aeruginosa*-induced increase in glucose permeability and hyperglycemia-induced growth [13]. In this study, our work indicates that metformin regulates the innate immunity independent of AMPK(AAK-2) in *C. elegans*.

It is noteworthy that based on the immunomodulatory properties, certain antibiotics improve the long-term outcome of patients with some chronic diseases [48,49]. For example, azithromycin, a macrolide, has beneficial effects of the patients with chronic obstructive pulmonary disease and cystic fibrosis (CF) [50,51]. Therefore, using immunomodulatory properties to develop new anti-infective drugs is an effective way. However, a role for antibiotic action-like metformin in the control of immunity has not been reported. Here we show that a short, nontoxic treatment with metformin can activate the innate immunity in *C. elegans* and mice through the p38/PMK-1-dependent manner. Our work put forward an ancient and conserved mechanism that metformin protects worms and mice from infection by pathogens.

## Materials and methods

### Worm strains and cultivation

Worms were maintained and propagated under standard conditions as previously described [52,53]. The following nematode strains were obtained from the Caenorhabditis Genetics Center (CGC), which is funded by NIH Office of Research Infrastructure Programs (P40 OD010440): N2 Bristol wild-type, KU25 *pmk-1(km25)*, AU3 *nsy-1(ag3)*, AU1 *sek-1(ag1)*, CB1370 *daf-2(e1370)*, SD184 *mpk-1(n2521)*, MT1434 *egl-30(n686)*, RB911 *fshr-1(ok778)*, RB1468 *dkf-2(ok1704)*, SAL144(*K08D8.5p::gfp*), AY102 *pmk-1(km25)*;acEx102, NR222 *rde-1(ne219)*;kzIs9, NR350 *rde-1(ne219)*;kzIs20, TU3401 (*sid-1(pk3321)*V;uIs69V) for neuronal RNAi. The nematode strain for intestinal RNAi *(sid-1(qt9)*; Is [vha-6pr::sid-1]; Is [sur-5pr:: GFPNLS]) were kindly provided by Dr. Gary Ruvkun (Massachusetts General Hospital, Harvard Medical School, Boston, MA). *C. elegans* mutants were backcrossed three times into the WT strain (N2) and used in the laboratory.

### RNA interference

The strains of *E.coli*used for RNAi were obtained from the Ahringer library [54]. RNAi feeding experiments were performed on synchronized L1 to L2 larvae at 20°C. Briefly, *E. coli* strain HT115(DE3) expressing dsRNA was grown overnight in LB broth containing 100 μg/ml ampicillin at 37°C, and then spread to NGM plates containing 100 μg/ml ampicillin and 5 mM isopropyl 1-thio-β-D-galactopyranoside (IPTG). The RNAi-expressing bacteria were grown overnight at 25°C. Synchronized L1 to L2 larvae were placed on RNAi plates until they reach maturity at 20°C. *Unc-22* RNAi was included as a positive control in all experiments to account for RNAi efficiency.

### Infection assay

*E.coli* OP50, *S. enterica* SL1344, *E. faecalis* ATCC 29,212, and *P. aeruginosa* PA14 were grown overnight in LB broth at 37°C, and *S. aureus* NCTC8325 was grown overnight in tryptic soy broth (TSB, BD, Sparks, MD) at 37°C, were then spread to NGM plates. All infection assays were performed on NGM agar plates or NGM plates supplemented with or without 50 mM of metformin. Synchronized populations of worms were cultivated on *E.coli* OP50 at 20°C until the young adult stage (i.e., within 12 h beyond the L4 stage). About 50–60 worms were transferred to NGM agar plates containing *S. enterica* SL1344, *S. aureus* NCTC8325, and *P. aeruginosa* PA14 at 25°C, respectively. The number of living worms was counted at 12 h intervals. Immobile adult worms unresponsive to touch were scored as dead. Three plates were tested per assay and all experiments were performed three times independently.

### Fluorescence microscopy

Synchronized L1 worms of the K08D8.5::GFP strain were transferred to agar plates supplemented with or without 50 mM of metformin. The images were obtained by using a Zeiss Axioskop 2 plus fluorescence microscope (Carl Zeiss, Jena, Germany) with a digital camera. Fluorescence intensity was quantified by using the ImageJ software (NIH). Three plates of about 40 animals per plate were tested per assay and all experiments were performed three times independently.

### Quantitative real-time PCR

Total RNA was extracted from worms with TRIzolReagent (Invitrogen) as previously described [55]. Random-primed cDNAs were generated by reverse transcription of the total RNA samples with SuperScript II (Invitrogen) and qPCR analysis was conducted by using SYBR Premix-Ex TagTM (Takara, Dalian, China) on an Applied Biosystems Prism 7000 Sequence Detection System (Applied Biosystems, Foster City, CA). Using *pmp-3* for an internal control as previously described [55]. The primers used for PCR were listed in Table S1.

### Quantification of intestinal bacterial loads

Synchronized populations of worms were cultivated on *E.coli* OP50 at 20°C until the young adult stage. *P. aeruginosa*/GFP were grown in LB liquid medium containing ampicillin (100 μg/ml) at 37°C overnight and plated onto NGM plates. Worms then were transferred to NGM agar plates (supplemented with or without 50 mM of metformin) containing *P. aeruginosa*/GFP for 48 h at 25°C [39]. To eliminate the *P. aeruginosa*/GFP around the surface of worms, worms were transferred to NGM agar plate seeded with *E. coli*OP50 for 15 min for three times [39]. Ten worms were transferred into 50-µl PBS plus 0.1% Triton and ground [39]. The lysates were serially diluted by 10-folds in sterilized water and spread onto LB agar plates/ampicillin at 37°C. After 1 d of incubation at 37°C, colonies of *P. aeruginosa*/GFP were counted. Five plates were tested per assay and all experiments were performed three times independently.

### Western blotting

After worms and lung tissues were homogenized in liquid nitrogen, the homogenate was lysed on ice for 60 min in lysis buffer (BioTeKe). The lysates of total protein were loaded (40 μg per well) and separated on a 10% SDS polyacrylamide gel. Proteins were then transferred to immobilon-PSQ transfer PVDF membrane (Millipore, Bedford, MA). Phosphorylated PMK-1 protein was detected using an anti-active p38 polyclonal antibody from rabbit (1:1000 dilution; Abcam, ab4822), and anti-beta-actin antibodies (1:1000 dilution; Abcam, ab227387). The secondary antibody was a peroxidase-coupled anti-rabbit IgG (1:20,000 dilution; Abmart). Blots were developed using the Super Signal chemiluminescence substrate (Pierce). Band intensities were measured using ImageJ software.

### Animal studies

C57BL/6 mice were inoculated with *P. aeruginosa*-laden agarose beads, as previously described [56]. The average 50 µl agar-beads suspension contained 1.0 × 10^6^ CFUs/mouse. The agar-beads suspension was ready for inoculation in the lungs of mice by an intratracheal injection. Meanwhile, some of animals received daily doses of 200 mg/kg body weight metformin (#D150959‑5G) and SB202190 (20 μg/kg/d) through intraperitoneal injection for 5 d.

### Statistics

Differences in survival rates were analyzed using the log-rank test. Differences in gene expression and fluorescence intensity were determined by performing a one-way ANOVA followed by a Student-Newman-Keulstest. Data were analyzed using the SPSS17.0 software (IBM, Armonk, New York).
