# Supplementary material for: Metformin promotes innate immunity through a conserved PMK-1/p38 MAPK pathway
Source: Virulence. 2019 Dec 28;11(1):39–48. doi: 10.1080/21505594.2019.1706305 (PMC6961722; doi:10.1080/21505594.2019.1706305)
Supplement: Supplemental Material [file kvir-11-01-1706305-s001.docx]

**
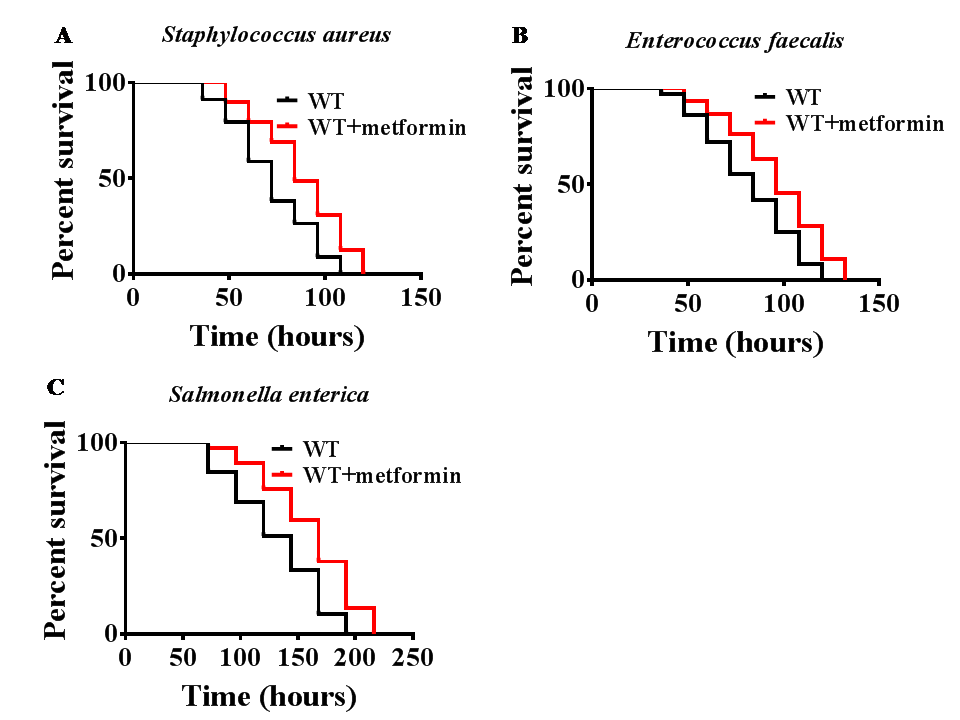
**

**Figure S1 metformin (50 mM) enhances the resistance to *S. aureus* (A), *E. faecalis* (B) and *S. enterica* (C) infection in *C.elegans*.** *P* < 0.001(log-rank test), relative to WT.


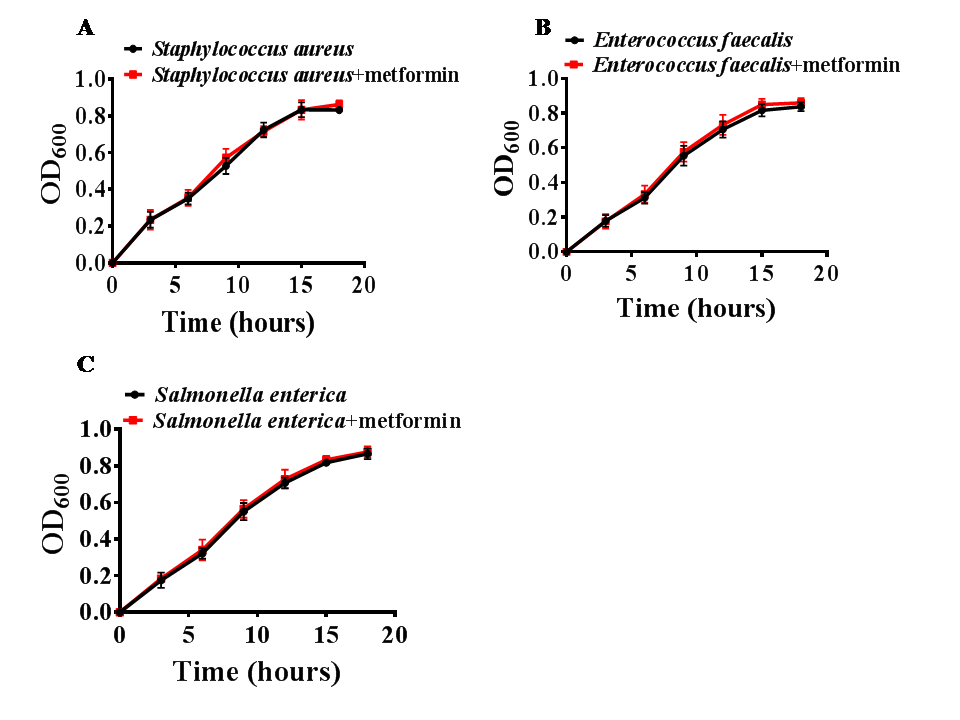


**Figure S2 metformin (50 mM) did not inhibit the proliferation of *S. aureus* (A), *E. faecalis* (B), *S. enterica* (C).**


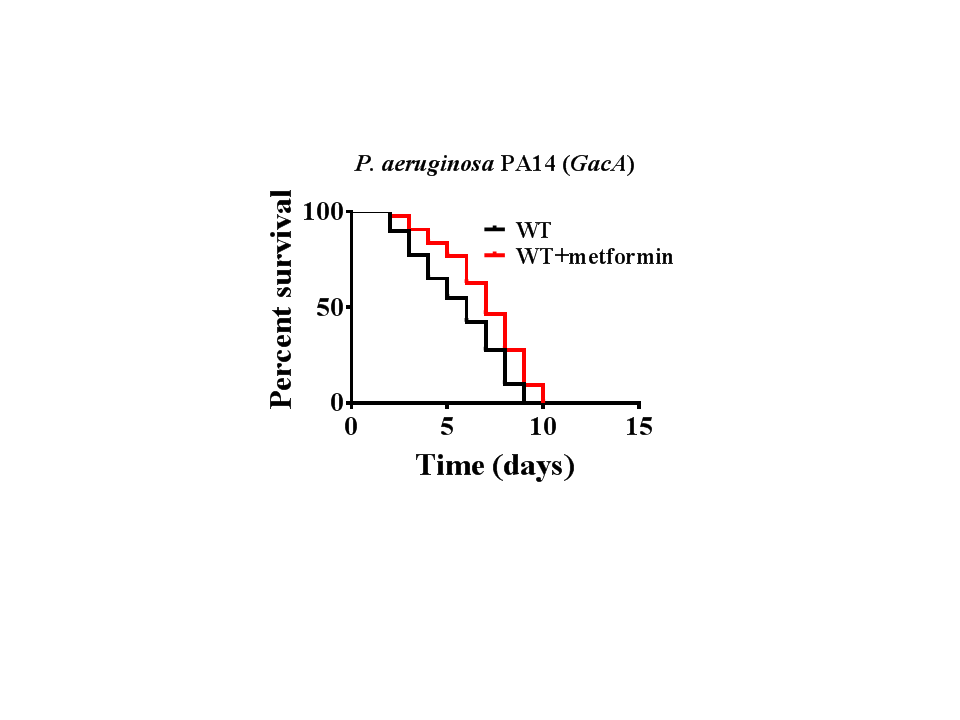


**Figure S3 metformin (50 mM) exhibited increased resistance to *P. aeruginosa* (PA14 *GacA* mutant) in *C.elegans*.** *P* < 0.05(log-rank test), relative to WT.


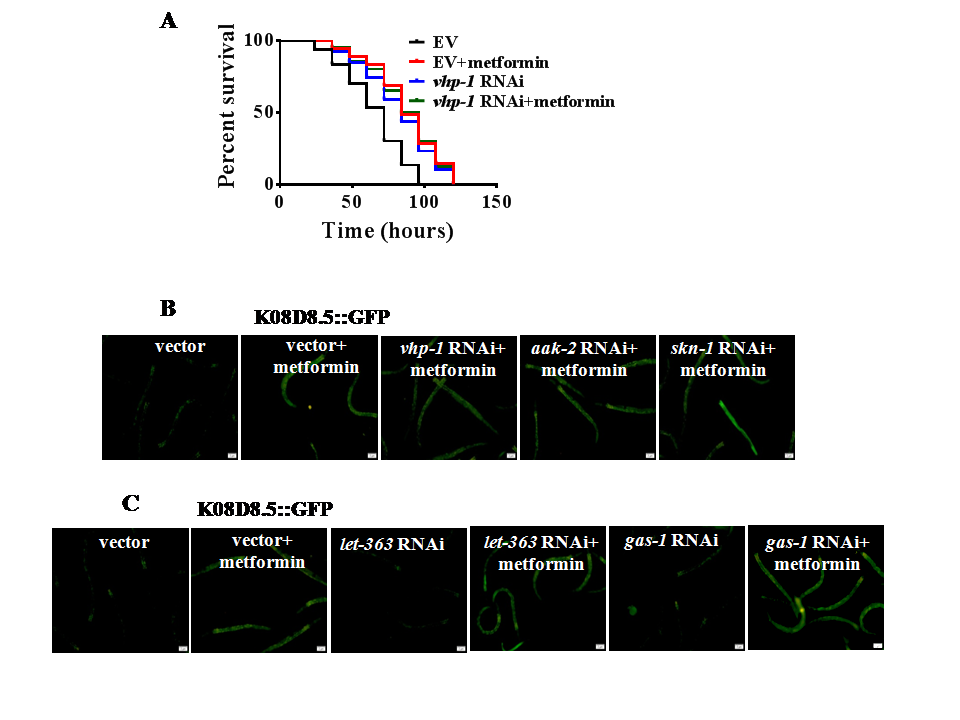


**Figure S4 *Vhp-1*RNAi was non-additively beneficial with metformin (50 mM) (A).** *P* = 0.358(log-rank test), relative to *vhp-1* RNAi. **knockdown of *vhp-1*, *aak-2*, *skn-1*, *gas-1*, *let-363* did not influence the level of *K08D8.5::GFP* in metformin treated animals (B) and (C).**
